# The Witness-Aimed First Account (WAFA): A new technique for interviewing autistic witnesses and victims

**DOI:** 10.1177/1362361320908986

**Published:** 2020-03-13

**Authors:** Katie Maras, Coral Dando, Heather Stephenson, Anna Lambrechts, Sophie Anns, Sebastian Gaigg

**Affiliations:** 1 University of Bath, UK; 2 University of Westminster, UK; 3 City, University of London, UK; 4 University of Sussex, UK

**Keywords:** autism, Criminal Justice System, event segmentation, interviewing, memory, narratives, police, support, victim, witness

## Abstract

Autistic people experience social communication difficulties alongside specific memory difficulties than impact their ability to recall episodic events. Police interviewing techniques do not take account of these differences, and so are often ineffective. Here we introduce a novel Witness-Aimed First Account interview technique, designed to better support autistic witnesses by diminishing socio-cognitive and executive demands through encouraging participants to generate and direct their own discrete, parameter-bound event topics, before freely recalling information within each parameter-bound topic. Since witnessed events are rarely cohesive stories with a logical chain of events, we also explored witnesses’ recall when the narrative structure of the to-be-remembered event was lost. Thirty-three autistic and 30 typically developing participants were interviewed about their memory for two videos depicting criminal events. Clip segments of one video were ‘scrambled’, disrupting the event’s narrative structure; the other video was watched intact. Although both autistic and typically developing witnesses recalled fewer details with less accuracy from the scrambled video, Witness-Aimed First Account interviews resulted in more detailed and accurate recall from autistic and typically developing witnesses, for both scrambled and unscrambled videos. The Witness-Aimed First Account technique may be a useful tool to improve autistic and typically developing witnesses’ accounts within a legally appropriate, non-leading framework.

**Lay abstract:**

Autistic people may be more likely to be interviewed by police as a victim/witness, yet they experience social communication difficulties alongside specific memory difficulties that can impact their ability to recall information from memory. Police interviewing techniques do not take account of these differences, and so are often ineffective. We developed a new technique for interviewing autistic witnesses, referred to a Witness-Aimed First Account, which was designed to better support differences in the way that autistic witnesses process information in memory. The Witness-Aimed First Account technique encourages witnesses to first segment the witnessed event into discrete, parameter-bound event topics, which are then displayed on post-it notes while the witness goes onto freely recall as much information as they can from within each parameter-bound topic in turn. Since witnessed events are rarely cohesive stories with a logical chain of events, we also explored autistic and non-autistic witnesses’ recall when the events were witnessed in a random (nonsensical) order. Thirty-three autistic and 30 typically developing participants were interviewed about their memory for two videos depicting criminal events. Clip segments of one video were ‘scrambled’, disrupting the event’s narrative structure; the other video was watched intact. Although both autistic and non-autistic witnesses recalled fewer details with less accuracy from the scrambled video, Witness-Aimed First Account interviews resulted in more detailed and accurate recall from both autistic and non-autistic witnesses, for both scrambled and unscrambled videos. The Witness-Aimed First Account technique may be a useful tool to improve witnesses’ accounts within a legally appropriate, non-leading framework.

Autism spectrum disorder (ASD) is characterised by persistent difficulties in social communication and restricted and repetitive behaviours and interests (American Psychiatric Association [APA], 2013). Core characteristics of ASD, such as difficulty in gauging social interactions and understanding the thoughts and intentions of others, have been linked to an increased risk of coming into contact with police as a victim/witness^1^ or suspect (e.g. Brown-Lavoie et al., 2014; Chaplin & Mukhopadhyay, 2018; Heeramun et al., 2017; Lindblad & Lainpelto, 2011; Rava et al., 2017; Tint et al., 2017, 2019; Weiss & Fardella, 2018). However, knowledge is currently limited regarding how best to interview autistic individuals in order to elicit the most complete and accurate information about what they have experienced.

The prevalent approach for collecting witness information from adults in England and Wales is to conduct a face-to-face interview, using a phased approach that commences with a free narrative account of what has occurred, followed by a series of more probing questions concerning the topics verbalised during that free recall (e.g. Milne & Bull, 1999; Ministry of Justice, 2011). This approach is deemed important to ensure that memory for the event is uninfluenced by the interviewer. It is ineffective, however, for autistic individuals, who present with a distinct memory profile whereby episodic memory is typically reduced (see Gaigg & Bowler, 2018), particularly on tasks requiring a free narrative account of experienced events (Adler et al., 2010; Bowler et al., 1997, 2008; Chaput et al., 2013; Crane et al., 2009, 2010, 2012; Crane & Goddard, 2008; Goddard et al., 2007; Smith et al., 2007; Tanweer et al., 2010). Autistic individuals also often experience source monitoring difficulties on unsupported free recall tests; for example, in recollecting when, where or with whom an event occurred (e.g. Bennetto et al., 1996; Bowler et al., 2004, 2008; Cooper et al., 2016; Lind & Bowler, 2009; Maras et al., 2013). Thus, when questioned using a free narrative approach, a growing body of evidence suggests that autistic witnesses typically recall significantly less information about experienced events than typically developing (TD) individuals (e.g. Almeida et al., 2019a; Bruck et al., 2007; Henry et al., 2017; Maras & Bowler, 2010, 2011, 2012a; Maras et al., 2012; M. Mattison et al., 2018, 2015; McCrory et al., 2007), and often with less accuracy (Maras & Bowler, 2010, 2011; Maras et al., 2012, 2013).

Difficulties in encoding and/or retrieving the *relations* among items of experience has been suggested to underlie these episodic memory difficulties in autism (see, for example, Bowler et al., 2011, 2014; Cooper & Simons, 2019; Gaigg & Bowler, 2018; Gaigg et al., 2008, 2015). For example, autistic people make more familiarity-based recognition judgements (which can be mediated on the basis of available item-specific information alone) and fewer autonoetic recollective-based responses, which require drawing on context and the relations among contextual details to aid remembering (e.g. Bowler et al., 2000, 2004, 2007, 2008; Cooper et al., 2015; Lind & Bowler, 2010; Meyer et al., 2014; Tanweer et al., 2010; see also Barnes & Baron-Cohen, 2012). Evidence from studies of free recall also suggests that autistic individuals have difficulties drawing on relationships among items, but not item-specific meaning, to facilitate recall (e.g. Gaigg et al., 2008).

Beyond experimental memory paradigms, most autistic people also experience some degree of difficulty in their ability to construct and relate a coherent narrative (Tager-Flusberg et al., 2005). While generally not differing from language-matched TD individuals on basic aspects of narrative, such as the identification of the main elements of an event (Beaumont & Newcombe, 2006; Capps et al., 2000; Hilvert et al., 2016; Hogan-Brown et al., 2013; Losh & Capps, 2003; Tager-Flusberg & Sullivan, 1995), autistic individuals’ narratives often lack causation and coherence, particularly with regards to temporality and the causal connection of plot points (e.g. Capps et al., 2000; Diehl et al., 2006; Hilvert et al., 2016; King et al., 2014; Kuijper et al., 2017; Lee et al., 2018; Losh & Capps, 2003, 2006; Losh & Gordon, 2014; McCabe et al., 2013; Tager-Flusberg, 2000). It has been suggested that these narrative difficulties may be explained in part by difficulties in considering the needs and perspectives of the listener (e.g. Baron-Cohen, 1988; Bruner & Feldman, 1993; Colle et al., 2008; Goldman, 2008; Hilvert et al., 2016; Tager-Flusberg, 1995; Tager-Flusberg & Sullivan, 1995) as well as in generating, strategically planning and organising one’s recall of an event (Barnes & Baron-Cohen, 2012).

Critically, autistic individuals can utilise the relations among items and produce narratives of a similar quality to TD individuals when the task is structured in a manner that enables the person to organise their responses (e.g. Bowler et al., 1997, 2000, 2008; Hermelin & O’Connor, 1970; Losh & Gordon, 2014; Tager-Flusberg, 1991). For example, when test procedures involve cued and directed recall or recognition retrieval questioning techniques, autistic individuals’ performance is often equivalent to that of TD comparison participants (e.g. Bennetto et al., 1996; Bowler et al., 1997, 2004, 2008, 2015; Hare et al., 2007; Maras & Bowler, 2011; Maras et al., 2012, 2013; Yamamoto & Masumoto, 2018; Zalla et al., 2010). This pattern of memory performance in ASD suggests that difficulties arising during spontaneous memory retrieval can be compensated through appropriate scaffolds, which has led to the formulation of the Task Support Hypothesis (Bowler et al., 1997, 2004), positing that memory performance in ASD is enhanced on tasks that provide more support for the to-be-remembered material at test.

As well as providing specific support for relational processing, cued recall and recognition tests may also be effective in supporting ASD retrieval difficulties by reducing demands on executive processes, freeing up cognitive resources required to elicit an appropriate search strategy and generate a response (Maister et al., 2013). This is particularly pertinent because autistic individuals often rely on effortful executive resources as a compensatory mechanism for diminished relational memory in order to retrieve episodic and relational memories (Goddard et al., 2014; Maister et al., 2013), yet they also often experience broad difficulties in executive functioning (see Demetriou et al., 2018). More directive prompting further serves to diminish the implicit social demands and ‘open-endedness’ of the task (Kenworthy et al., 2008; Ozonoff, 1995; White, 2013; White et al., 2009). These findings are important for the development of theoretically driven interviewing techniques to improve autistic witnesses’ testimony.

However, while cued recall, closed and directed questioning, and recognition questioning techniques may be effective for supporting autistic witnesses to recall more information in laboratory settings, the use of questions that are not preceded by a witness-led account is unacceptable for the purposes of the Criminal Justice System (CJS) for several reasons. First, the questions would be solely guided by what information the *interviewer knows* at the time (e.g. from other witnesses, crime reports, etc.) and, relatedly, what the *interviewer thinks* is important, rather than the full gamut of information witnesses have actually experienced. Second, witnesses tend to produce less information in response to more specific questions as opposed to free recall prompts (e.g. Fisher & Geiselman, 1992; Milne & Bull, 1999), thus reducing the number of topics that can be *safely* explored using cued, directed, closed and recognition-type questions. Third, specific questions can lead witnesses and introduce demand characteristics which can reduce accuracy and increase errors (Fisher & Geiselman, 2010). In the case of autistic witnesses, this is particularly concerning because, although research indicates that they are not more suggestible to memory distortions (Bruck et al., 2007; Maras & Bowler, 2011, 2012b; McCrory et al., 2007; North et al., 2008), they can be more compliant and prone to guessing when pushed (Chandler et al., 2019; North et al., 2008; but see Maras & Bowler, 2012b).

Several studies have investigated different techniques for supporting autistic witnesses within a phased interview approach, but none have been shown to be effective in increasing both the amount and accuracy of reported details. Thus, there currently exists no empirically and theoretically driven model for interviewing autistic witnesses. Maras and Bowler (2010) investigated the cognitive interview (CI; Fisher & Geiselman, 1992) – a widely used, evidence-based police interviewing model that has been shown to increase the amount of correct information that witnesses recall without a concomitant increase in errors (for a meta-analysis, see Memon et al., 2010) – but found it to be less effective for autistic adults. Not only did the CI (which includes a ‘mental reinstatement of event context’ technique) fail to elicit more details from autistic witnesses compared to a structured comparison interview (which had the same number of retrieval attempts and follow-up questions but without the cognitive mnemonics), it also resulted in significantly more errors and so reduced the overall accuracy of the information that autistic witnesses recalled compared to TD witnesses. Sketching to reinstate the context at interview (Sketch-RC) is a recently developed variant of the CI which has been found to support witnesses from various populations (e.g. TD adults, older adults, TD children) to recall more information without concomitant increases in errors, and in some cases with significantly reduced errors (e.g. Dando, 2013; Dando et al., 2009, 2011). Here, witnesses are supported to construct a narrative by asking them to sketch the event while verbally describing what they are drawing (see Dando, 2013). Mattison et al. (2018) and Mattison et al. (2015) found that while sketching significantly improved the accuracy of recall of an episodic event in autistic children and adolescents versus a matched group who were unsupported, it did not increase the number of correct details reported by autistic participants, indicating a need for research to explore further techniques.

Given the relevant literature, it is sensible to assume that the memory performance of autistic witnesses may be mediated by interview structure. Most of the current best practice methods, such as the CI, and also the Sketch-RC technique, all rely on an unbounded free narrative recall to commence the interview and to scaffold the questioning that follows. For autistic witnesses, however, a lack of explicit parameters concerning what they are being asked to recall may be problematic (see, for example, White, 2013). Autistic witnesses are likely to be better supported at retrieval if more specific guidance were offered (see Bowler et al., 2004) alongside directive prompts (e.g. Losh & Capps, 2003) that are nonetheless non-leading and protect the integrity of the information (should criminal proceedings commence at any point thereafter). Here we consider how to support autistic witnesses to provide a free narrative within an evidence-based and legally appropriate verbal interview protocol aimed at eliciting a detailed account of an experienced event.

Adaptations to interview protocols must take account of the way in which autistic individuals perceive, process and retrieve information, as well as the limited or distorted viewing conditions that are often experienced when witnessing events in real life (Memon et al., 2003). According to event segmentation theory (Zacks et al., 2001; Zacks & Swallow, 2007), in undistorted, uninterrupted viewing conditions, incoming perceptual information is typically automatically segmented into discrete and distinct meaningful event components. This event segmentation is crucial for action comprehension and provides a structure for later memory, facilitating the ease with which items from a particular segment of an event are recalled (e.g. Swallow et al., 2009). For example, people tend to segment events when there are points of change, such as in location, or in actors’ positions, movements, goals or intentions (e.g. Hard et al., 2006; Newtson et al., 1977; Speer et al., 2007; Zacks, 2004). Such ‘chunking’ enables the viewer to maintain compact representations of extended sequences of acts on-line by decreasing working memory demands, which facilitates the storing of the information in long-term memory for later retrieval (Ongchoco & Scholl, 2019; Sirigu et al., 1995; Zacks et al., 2001). However, autistic individuals may interpret and extract meaning from events differently, which in turn may impact their recall of them (e.g. Berna et al., 2016; Crane et al., 2010; Loveland et al., 1990; Williams et al., 2006). For example, Zalla et al. (2013) reported that autistic participants had more difficulty identifying event boundaries than TD individuals, which was associated with diminished event recall and poorer memory for event sequences. Together these findings indicate that autistic individuals may not spontaneously utilise an event’s naturally occurring segments and breakpoints to scaffold their memory and retrieval to the same extent as TD individuals, which may be related to differences in the way that event information is encoded and organised (see also Miller et al., 2014). In the context of recalling witnessed events, this may manifest as a greater difficulty in spontaneously generating a complete narrative of the event, placing greater demand on executive processes (see Maister et al., 2013), which in turn may negatively impact upon the quantity (completeness) and/or quality (accuracy) of information recalled.

## The current study

There is a clear need for an interviewing model that supports an autistic witness’ individual processing style while utilising parameter-bound retrieval methods, but this must be compatible with both practical frameworks (e.g. *Achieving Best Evidence* guidance; Home Office, 2011) and theoretical understanding of the importance of witness-compatible retrieval (see Fisher & Geiselman, 1992). It must also be beneficial for non-autistic witnesses in order to be of practical value. The aim of the present study was twofold. First, to empirically test a novel interviewing technique whereby the witness self-segments their memory of an event into their own discrete parameter-bound ‘topic boxes’ at the outset, before engaging in an exhaustive free recall retrieval attempt (followed by interviewer probing) within the parameters of each topic box in turn. Given that free recall is problematic for autistic individuals, more supportive and witness-compatible interviewing of this nature that provides a frame of reference for the event and its component parts should help. In this novel method, which we refer to as a Witness-Aimed First Account (WAFA) interview, the witness self directs their recall, as would happen during a typical free narrative account, but rather than having a free flow verbalization of the entire event (which is difficult for autistic individuals) they provide their own segmentation of the event. The topic boxes are displayed on post-it notes as a reminder of the structure of the event, reducing demands on executive processes and allowing the witness to focus their search and retrieval strategies within individual segments. In addition to quantitative measures of participants’ recall under WAFA versus control interviews, we also sought qualitative feedback from participant witnesses regarding their perceived utility of the different interview techniques.

Second, we examined whether autistic individuals would be relatively less affected than TD individuals when an event has a weak narrative structure – as is often the case in real life where only partial event information is perceived under poor viewing conditions, or when viewing is interrupted. Here, it was predicted that TD witnesses’ recall would appear more similar to autistic witnesses since they too would find it more difficult to generate a narrative. In order to test this, participants viewed two videoed events – one of which was ‘scrambled’ in 4–5 s segments that cut through the event’s natural breakpoints or borders (see Schwan et al., 2000; Schwan & Garsoffky, 2004; Swallow et al., 2009) – and the other was viewed intact.

Based on the Task Support Hypothesis and relevant empirical literature, we predicted that WAFA interviews would elicit more detailed and accurate accounts from both autistic and TD mock witnesses. We also expected a diminution in both the completeness and accuracy of recall when the event’s narrative is scrambled (compared to when it is intact) for both autistic and TD witnesses, but that this difference would be somewhat attenuated for the autistic group and when interviewed with the WAFA model.

## Method

### Design

The study employed a 2 (Group: ASD vs TD) × 2 (Interview: WAFA vs Control) × 2 (Video: Scrambled vs Unscrambled) mixed design, where Video was within participants (counterbalanced between the two videos, groups and interview conditions). All participants watched two videos, one of which was scrambled, and were interviewed about each video with either a WAFA interview or control interview. The dependent variable was interview performance, measured by the number of correct and incorrect details reported, and overall accuracy scores (correct details as a function of total details recalled). Immediately following the final interview, each participant completed a questionnaire designed to collect quantitative and qualitative data concerning their interview experience.

### Participants

A power analysis using G*Power3.1 (Faul et al., 2007) indicated that a total sample size of 62 would give 90% power to detect a medium-to-large effect of group and interview type (i.e. to have significant implications for practice). A total of 63 participants were recruited: 33 autistic adults (27 males) and 30 TD adults (16 males). Autistic participants were recruited through existing databases at the University of Bath and City, University of London, and through ongoing recruitment calls for new participants via social media, local autism networks and organisations and local newspaper advertisements. All autistic participants had received a formal diagnosis of ASD by experienced clinicians through the UK’s National Health Service according to *Diagnostic and Statistical Manual of Mental Disorders* (4th ed.; *DSM*-IV; APA, 2000) or *DSM*-5 criteria (APA, 2013), which was confirmed with a copy of their original detailed diagnostic report. Those who had received a diagnosis but were unable to produce a detailed letter received the Autism Diagnostic Observation Schedule, Second Edition (ADOS-2; Lord et al., 2012), to confirm their diagnoses.

TD participants were recruited through social media, local newspaper advertisements and existing contacts and databases. In order to screen for possible undiagnosed ASD, all TD participants completed the Autism-Spectrum Quotient (AQ; Baron-Cohen et al., 2001), and the sample all scored below the recommended cut off of 32 points (Woodbury-Smith et al., 2005). As expected, the ASD group scored significantly higher than TD participants on the AQ, *t*(61) = 10.36, *p* < 0.001, *d* = 2.63. Specific data on ethnicity, socioeconomic status and educational attainment levels were not recorded.

Participants completed Vocabulary and Matrix Reasoning subtests from the Wechsler Adult Intelligence Scale (WAIS-IV; Wechsler, 2008) as indices of verbal and non-verbal ability on which groups were matched. Participants also completed three working memory subtests from the WAIS IV: Digit Span, Arithmetic and Letter–Number Sequencing, partly to serve as filler tasks between videos and interviews, and partly to establish whether autistic and TD groups differed on a measure of executive function that might be relevant to retrieving complex events. The sum of the standardised scores across the three working memory measures was used as an index of working memory. A series of two-way analyses of variance (ANOVAs) indicated that there were no main effects of Group, Interview or Group × Interview interactions for age (all *p*s > 0.156, $\eta_{p}^{2}s<0.03$), vocabulary (all *p*s > 0.304, $\eta_{p}^{2}s<0.02$), matrix reasoning (*p*s > 0.138, $\eta_{p}^{2}s<0.04$) or working memory index scores (all *p*s > 0.515, $\eta_{p}^{2}s<0.01$) (Table 1).

**Table 1.** Age and vocabulary, matrix, and AQ scores for the ASD and TD groups within each interview condition (standard deviations are in parentheses).

	ASD (*n* = 33)	TD (*n* = 30)
WAFA (*n* = 31)	(*n* = 16)	(*n* = 15)
Age	34.10 (10.77)	37.88 (13.74)
Vocabulary	11.50 (2.68)	12.00 (1.69)
Matrix	13.06 (3.36)	12.93 (2.74)
Working memory index	34.97 (8.42)	33.07 (7.86)
AQ	34.19 (7.79)	15.40 (7.17)
Control interview (*n* = 32)	(*n* = 17)	(*n* = 15)
Age	35.87 (7.81)	41.78 (15.74)
Vocabulary	10.94 (2.70)	12.07 (2.69)
Matrix	11.19 (3.62)	12.20 (3.82)
Working memory index	32.19 (10.69)	32.71 (10.04)
AQ	34.00 (8.41)	14.80 (5.72)

WAFA: Witness-Aimed First Account; ASD: autism spectrum disorder; TD: typically developing; AQ: Autism-Spectrum Quotient.

Participants were reimbursed for their time at standard University rates. The study received ethical approval from the Psychology Ethics committees at the University of Bath (16-026) and City, University of London (PSYETH (S/L) 15/16 210).

### Crime stimulus videos

Two videos were developed specifically for the purposes of this study.^2^ One depicted a handbag theft in a car park and the other a fight in a bar, and each video lasted around 1 min 40 s. The video of the handbag theft began with three friends chatting as they walked towards a car in a carpark. After getting in and driving off they spotted another friend walking along and stopped to offer her a lift. Just after she got in the car a young male knocked on the window and began to ask for directions, before reaching in through the open window, grabbing the handbag from the lap of the front passenger and running off. The front passenger got out of the car and ran after him. In the bar fight video, a male was buying drinks at the bar for a female friend, while another female walked over to chat about a coursework assignment. On getting their drinks the male and female walked over to the other side of the bar where they sat down at a table. Their conversation was interrupted by two males talking in raised voices that escalated into shouting. One of the males pushed the other before punching him to the ground and repeatedly punching him twice more. The male friend went over and declared that he was unconscious, while a girl who was sitting behind them called an ambulance. The bar fight and car park theft videos were designed to be broadly similar in terms of number and range of details. For example, each video utilised six actors (all aged between 18 and 30 years) plus bystanders, portrayed a similar number of key actions before and during the crimes, and comprised visually rich surroundings with additional person, object and surrounding details available. There was no difference in the number of correct details that participants reported between the bar fight (max. = 213 reported correct details) or car park theft videos (max. = 209 reported correct details), *F*(1, 60) = 0.25, *p* = 0.617, $\eta_{p}^{2}=0.004$.

Two versions of each video were created: one with an ‘unscrambled’ (intact) narrative and the other a ‘scrambled’ narrative where the event’s natural event boundaries and narrative coherence (story) was disrupted. This was determined during a pilot study in which 41 participants indicated where they perceived each video’s natural event boundaries to start and finish. Response frequencies were then plotted on a time graph, and 4–5 s segments of the video were selected that cut across these natural event boundaries. Videos were then reconstructed by placing these clip segments in a random order, thus removing each video’s natural segmentation and narrative structure to form scrambled versions.

### Procedure

Participants were tested individually in dedicated laboratory space at the University of Bath or City, University of London. After watching the first video participants completed unrelated tasks (including WAIS subtests) for approximately 30 min, before they were interviewed about the video under their assigned interview condition (WAFA vs control). Following a break, they watched the second video, followed by unrelated tasks (the remaining WAIS subtests) again taking around 30 min, before they were interviewed for their memory of the second video (using the same assigned interview condition as before). The order in which the videos were presented and whether the handbag theft or bar fight was scrambled was counterbalanced between participants and interview conditions.

#### Interviews

The WAFA interview procedure was developed specifically for this research by the first two authors. All interviews were conducted by one of three female research assistants who were trained in accordance with the UK investigative interview model (PEACE) and Achieving Best Evidence guidance (Home Office, 2011) by the second author. Interviews in both conditions were preceded by a rapport phase in which the interviewer engaged in conversation with the participant about a neutral topic of interest, such as whether they had taken part in research before, and then an ‘engage and explain’ phase where the interviewer outlined the purpose and structure of the interview (which differed for control and WAFA interviews – see below for details). Participants were informed that the interviewer had not seen the video themselves and that they should therefore describe the event in as much detail as possible. They were instructed to recall everything that they could remember, even if only partial pieces of information came to mind, but not to guess.

*Control interviews* then asked participants to engage in an exhaustive and uninterrupted free recall attempt of the entire video. After the witness had indicated that they had come to the end of their free recall attempt they were then asked follow-up witness-compatible tell/explain/describe questions that probed the witness’ initial account in more detail. If the witness did not refer to an event or action they were not questioned about it; however, if they recalled that ‘a guy was knocked out’ they would be probed for further details of this (how, who, where, when, etc.) adopting the same language that was used by the witness (e.g. ‘describe the guy who got knocked out’).

**Figure 1.**
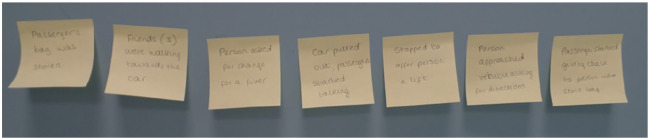
Example of self-segmentation of recall by a participant in phase 1 of the WAFA interview condition.

*WAFA interviews* asked witnesses to self-segment their free narrative recollection from the beginning. This was achieved through asking the witness: ‘In just a couple of sentences or a few words, what was the most important event that happened in the video’. The interviewer noted down the event on a post-it note which was then displayed on the wall adjacent to the desk and visible to both interviewer and witness. They were then thanked and informed that the interviewer would return to that event in a short while. They were then asked, ‘tell me something else that happened’, which was again noted and displayed on a post-it note. This continued until the participant indicated that they had completed segmenting the events (see Figure 1 for an example). Once complete, the interviewer then revisited each of the self-directed free narrative topics in turn, and in the order that the witness recalled them, asking the witness to provide a free recall account within that topic. This was then followed by tell/explain/describe questions probing further detail about each event with the same witness compatible-questioning used in the control interviews.

All interviews concluded with a closure phase, in which the participant was thanked for their time and asked if they would like to add to or change their account or if they had any questions.

#### Post-interview questionnaire

A paper-based post-interview perceptions questionnaire was devised for this study. The questionnaire comprised a total of 11 questions, of which 2 were open-ended, 6 were Likert-type questions, 2 were dichotomous (yes/no) and 1 offered a three-choice response (too fast/too slow/about right) (Appendix 1). Participants in both conditions completed 10 of the 11 questions, the final question (which asked about how useful they found the post-it notes) was completed by participants in the WAFA condition only.

### Interview coding

Interviews were transcribed, and then each unit of detail that participants recalled was coded as correct (if it matched that in the video) or incorrect (e.g. describing the perpetrator’s jumper as blue when in fact it was grey, or reporting an object that was not present in the video at all). Phrases were broken down and scored at the finest level of detail available. For example, a participant who reported ‘A friend got in [action] the woman’s [gender] car [object], wearing a brown [description] coat [clothing] and red [description] backpack [object]. Her [gender] name was Sarah [name] and she was headed into town [action]’ would receive 10 points (assuming that none of these details had already been mentioned previously). Accuracy scores were calculated by dividing the number of correct details reported by the total number of details (i.e. correct + incorrect details) reported. Items were only scored the first time they were mentioned, and statements that could not be verified or expressed opinion (e.g. ‘he looked a bit shifty’) were not coded. Twenty-three interviews (18.25%) were randomly selected and blindly recoded by an independent coder against the original videos. Strong agreement was reached between the raters, with intraclass correlation coefficients of 0.93 for correct details and 0.90 for incorrect details.

### Analysis plan and preliminary analyses

To examine the effects of group, interview technique and video narrative on witnesses’ recall performance, three 2 (Group: ASD vs TD) × 2 (Interview: WAFA vs Control) × 2 (Video: Scrambled vs Unscrambled) mixed ANOVAs (where Video was within participants) were conducted for correct details, incorrect details (errors) and overall accuracy scores, respectively. An alpha value of lower than 0.05 was used to indicate significant effects, and partial Eta squared ($\eta_{p}^{2}$) are reported throughout as estimates of effect sizes.

Inspection of the data revealed an outlier from the TD group as recalling an unusually high number of details (>3.5 *SD*s from the mean), and they were excluded from the analyses. Shapiro–Wilk tests of normality indicated that, with two exceptions, all dependent variables (i.e. correct details, errors and accuracy) were normally distributed in each Group × Interview × Video narrative condition combination (*p*s > 0.104). The two exceptions were as follows: (a) the autistic group’s accuracy scores in the control interview condition were negatively skewed for unscrambled videos; and (b) the TD group’s error scores in the WAFA condition were positively skewed for unscrambled videos (*p*s < 0.024). To correct this, square root transformations were applied to total error scores, while accuracy scores were reflected and square rooted. Analyses were run first with the original (untransformed) data and then again with the transformed data, and the pattern of findings remained the same. To aid interpretation of the data (e.g. regarding the absolute number of errors made) and because ANOVAs are considered to be fairly robust to deviations from normality (e.g. Schmider et al., 2010), findings from the untransformed data are reported below. Levene’s test for equality of variances indicated homogeneity of variances across all DVs (*p*s > 0.121).

## Results

Table 2 displays the number of correct details, errors and accuracy proportions of recall by autistic and TD witnesses within each interview and video narrative condition.

**Table 2.** Number of correct details, errors (incorrect details) and accuracy proportion scores as a function of group, interview condition and video narrative.

	ASD		TD	
	WAFA interview	Control interview	WAFA interview	Control interview
Unscrambled video				
Correct details				
Mean (*SD*)	125.41 (25.23)	113.97 (36.83)	144.97 (38.98)	129.04 (32.42)
Range	87–171	47–163	84.5–213	81–193
Errors				
Mean (*SD*)	13.13 (6.74)	17.56 (9.26)	15.47 (10.16)	17.79 (8.58)
Range	2–25	3–37	4–43	1–32.5
Accuracy score				
Mean (*SD*)	0.91 (0.04)	0.86 (0.04)	0.91 (0.04)	0.88 (0.06)
Range	0.81–0.98	0.73–0.96	0.83–0.96	0.77–0.99
Scrambled video				
Correct details				
Mean (*SD*)	100.72 (37.51)	82.91 (34.13)	116.97 (30.23)	99.00 (32.75)
Range	53.5–189	25–158.5	68.5–161	59–177
Errors				
Mean (*SD*)	15.34 (9.58)	18.74 (9.37)	19.07 (11.43)	20.04 (10.29)
Range	3–35	6–39	4–44	4–37
Accuracy score				
Mean (*SD*)	0.87 (0.07)	0.81 (0.09)	0.86 (0.06)	0.83 (0.08)
Range	0.77–0.96	0.61–0.93	0.78–0.96	0.70–0.96

WAFA: Witness-Aimed First Account; ASD: autism spectrum disorder; TD: typically developing; *SD*: standard deviation.

### Correct details

A 2 (Group: ASD vs TD) × 2 (Interview: WAFA vs Control) × 2 (Video: Scrambled vs Unscrambled) mixed ANOVA revealed a significant main effect of Group, *F*(1, 58) = 4.59, *p* = 0.036, $\eta_{p}^{2}=0.07$, with autistic participants recalling significantly fewer correct details (*M* = 105.33, *SD* = 31.86) than TD participants (*M* = 122.78, *SD* = 30.44) overall. There was also a main effect of Interview, *F*(1,58) = 4.08, *p* = 0.048, $\eta_{p}^{2}=0.07$, whereby, irrespective of Video or Group, more correct details were recalled in WAFA interviews (*M* = 121.73, *SD* = 30.91) compared to control interviews (*M* = 105.48, *SD* = 31.79). Finally, there was a main effect of Video, *F*(1, 58) = 61.88, *p* < 0.001, $\eta_{p}^{2}=0.52$. All participants recalled significantly fewer correct details from the scrambled video versions (*M* = 99.38, *SD* = 35.24) than the unscrambled video versions (*M* = 127.83, *SD* = 34.82).

**Figure 2.**
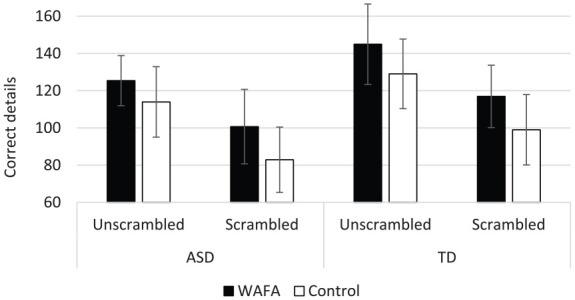
Correct details recalled for scrambled and unscrambled video narratives by autistic and TD groups in WAFA and control interviews (with 95% confidence error bars).

There were no Video × Group, *F*(1, 58) = 0.25, *p* = 0.875, $\eta_{p}^{2}<0.01$; Video × Interview, *F*(1, 58) = 0.34, *p* = 0.563, $\eta_{p}^{2}=0.01$; Group × Interview, *F*(1,58) = 0.02, *p* = 0.882, $\eta_{p}^{2}<0.01$; or Video × Group × Interview interactions, *F*(1, 58) = 0.09, *p* = 0.765, $\eta_{p}^{2}<0.01$ (Figure 2).

### Errors

There was a significant main effect of Video, *F*(1, 58) = 4.31, *p* = 0.042, $\eta_{p}^{2}=0.07$, with significantly more incorrect details recalled in videos which had a scrambled narrative (*M* = 18.23, *SD* = 10.07) compared to unscrambled videos (*M* = 15.96, *SD* = 8.76). There was no effect of Interview, *F*(1, 58) = 1.68, *p* = 0.200, $\eta_{p}^{2}=0.03$, or Group, *F*(1, 58) = 0.78, *p* = 0.380, $\eta_{p}^{2}=0.01$, and none of the Video × Group, *F*(1, 58) = 0.30, *p* = 0.584, $\eta_{p}^{2}=0.01$; Video × Interview, *F*(1, 58) = 0.29, *p* = 0.593, $\eta_{p}^{2}=0.01$; Group × Interview, *F*(1, 58) = 0.28, *p* = 0.599, $\eta_{p}^{2}=0.01$; or Video × Group × Interview interactions were significant, *F*(1, 58) = 0.01, *p* = 0.945, $\eta_{p}^{2}<0.01$ (Table 2).

### Accuracy

**Figure 3.**
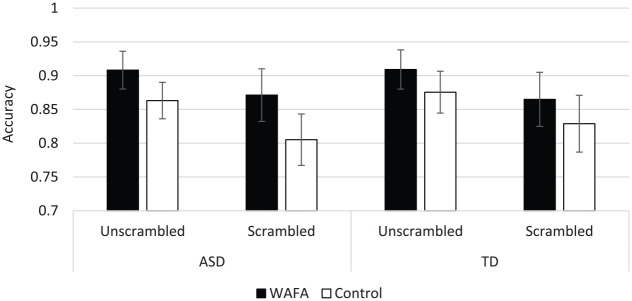
Accuracy of recall of scrambled and unscrambled videos by autistic and TD groups in WAFA and control interviews (with 95% confidence error bars).

There was a main effect of Interview, *F*(1, 58) = 8.56, *p* = 0.005, $\eta_{p}^{2}=0.13$, whereby participants were significantly more accurate in WAFA (*M* = 0.89, *SD* = 0.04) compared to control interviews (*M* = 0.84, *SD* = 0.07). There was also a main effect of Video, *F*(1, 58) = 34.34, *p* < 0.001, $\eta_{p}^{2}=0.37$. Participants were significantly less accurate in recalling videos that had a scrambled narrative (*M* = 0.84, *SD* = 0.08) than those which had an unscrambled narrative (*M* = 0.89, *SD* = 0.06). There was no main effect of Group, *F*(1, 58) = 0.25, *p* = 0.620, $\eta_{p}^{2}<0.01$; or significant Video × Group, *F*(1, 58) = 0.02, *p* = 0.897, $\eta_{p}^{2}<0.01$; Video × Interview, *F*(1, 58) = 0.54, *p* = 0.467, $\eta_{p}^{2}=0.01$; Group × Interview, *F*(1, 58) = 0.45, *p* = 0.504, $\eta_{p}^{2}=0.01$; or Video × Group × Interview interactions, *F*(1, 58) = 0.34, *p* = 0.563, $\eta_{p}^{2}=0.01$ (Figure 3).

#### Qualitative analysis of interviewee feedback

Questions 1 and 2 of the post-interview questionnaire asked all participants to explain what they liked about the interview (Q1), and what they did not like about the interview (Q2) using an open-ended invitation for each. Responses were analysed through a qualitative content analysis (Schreier, 2012) by a research assistant who was naïve to experiential design. Using an inductive data-driven approach, responses were open coded in the first instance, before being organised into categories. Meaning was then abstracted from the categories (Elo & Kyngäs, 2008), resulting in the emergence of a number of unique coding dimensions (primary codes) for each of the two questions. A random selection of 25% of the questionnaires were then coded by a second research assistant (who was also naïve to the experiential design) with reference to the first coder’s codebook, which listed all codes along with their definitions and examples of each code. Agreements and disagreements between both coders were tallied. Cohen’s Kappa was used to assess the level of agreement between the two coders, which revealed a high level of agreement, Kappa = 0.901, *p* = 0.002.

The primary codes that emerged for Question 1 concerned the procedure/structure of the interview, the social experience/environment and memory performance. For ease, we labelled all responses or comments regarding the interview structure and process as ‘interview’. Specific references to the interpersonal experience or the social context of the interview were coded as ‘social’, and references to perceived memory performance benefits were coded as ‘memory’ (see Table 3 for exemplar verbatim quotes).

**Table 3.** Exemplar quotes from post-interview feedback regarding what witnesses liked about the interview (participant group and interview condition are denoted in parentheses).

Interview
*I really liked the compartmentalized approach to getting what information I had retained from me* (ASD, WAFA)
*It was structured and the post it notes helped expand and elaborate* (ASD, WAFA)
*Post it notes created a helpful timeline* (ASD, WAFA)
*The bare details were taken quickly and then fleshed out later* (TD, WAFA)
*I liked it how we would go back to the post it notes which allowed me to remember crucial details* (TD, WAFA)
Social
*The woman was helpful and encouraging* (ASD, control interview)
*Interviewer was friendly and patient* (ASD, WAFA)
*It was a relaxed atmosphere* (TD, WAFA)
*I liked how informal it was, no pressure* (TD, control interview)
Memory
*Encouraged me to use my memory and to keep the memory in my head* (ASD, WAFA)
*Precise questions with opportunity to reflect helped my memory* (TD, WAFA)
*In-depth questions challenged my memory and helped me to think* (TD, WAFA)
*Allowed me to tell exactly everything I remembered, lead by me* (TD, control interview)
*The questions helped my memory* (TD, control interview)

ASD: autism spectrum disorder; WAFA: Witness-Aimed First Account; TD: typically developing.

Overall, 45.1% (*n* = 23) of participants stated that they liked the interview structure/procedure (Q1 code 1), 58.8% (*n* = 30) participants stated that they liked the interpersonal experience/social context (Q1 code 2), while 25.5% (*n* = 13) participants stated that they liked the memory benefits (Q1 code 3). Fisher’s exact tests revealed that significantly more participants in the WAFA interview condition liked the interview structure/process (62%) versus participants in the Control (25%), *p* = 0.016. There were no significant differences between conditions for the number of participants who reported liking the social context (WAFA: 46%; Control: 72%), *p* = 0.055, or who reported liking the memory performance benefits (WAFA: 36%; Control: 16%), *p* = 0.098.

The three primary codes that emerged for Question 2 concerned a positive response (that there was *nothing they did not like*), the procedure/structure of the interview and the social experience/environment. For ease, we labelled positive responses as ‘positive’. Negative comments regarding the interview structure and interview process (i.e. aspects of the procedure that were fixed) were labelled as ‘interview negative’, and specific references to not liking the interpersonal experience or the social context of the interview (i.e. how the witnesses felt in the presence of another person and in a situation that they could not control) were coded as ‘social negative’ (please see Table 4 for exemplar verbatim quotes).

**Table 4.** Exemplar quotes from post-interview feedback regarding what witnesses did not like about the interview (participant group and interview condition are denoted in parentheses).

Positive
*Nothing it was fine* (ASD, control interview)
*Nothing* (ASD, control interview)
*The interview was as it should have been* (TD, WAFA)
Interview negative
*I don’t enjoy the interrogation/communication part* (ASD, WAFA)
*It was a bit slow paced* (ASD, control interview)
*Being asked questions after being asked a question that was initially asked earlier as a prompt to elaborate* (ASD, control, interview)
*Repeating questions was tedious* (ASD, control interview)
Social negative
*‘The feeling of uncertainty’* (TD, control interview)
*Being filmed and recorded is very unnerving . . . I felt like a suspect rather than a witness* (TD, control interview)
*Being put on the spot* (TD, control interview)

ASD: autism spectrum disorder; TD: typically developing; WAFA: Witness-Aimed First Account.

Overall, 70.6% (*n* = 36) of participants made positive responses, typically stating that there was nothing they did not like about the interview (Question 2 code 1), 23.5% (*n* = 12) participants stated that they did not like the interview procedure/structure (Question 2 code 2), while just 5.9% (*n* = 3) participants stated that they did not like the interview environment (Question 2 code 3). Fisher’s exact tests revealed no significant differences across interview conditions for the number of participants who made positive responses (WAFA = 69.2%; Control = 72%), *p* = 0.599; interview negative comments (WAFA = 23.1%; Control = 24%), *p* = 0.536; or social negative comments (WAFA = 7.7%; Control = 4%), *p* = 0.515.

Five Likert-type questions (see Appendix 1) offered participants a range of response options, from 1 to 5 (where 1 = not at all/very uncomfortable to 5 = very useful/very comfortable/very well). Significant differences emerged between the WAFA and Control interview conditions for three of the five Likert-type questionnaire responses. Participants who were interviewed with the WAFA technique (*M* = 4.62, *SD* = 0.50) reported that the interview helped them to think harder than participants who received a Control interview (*M* = 4.04, *SD* = 0.68), *F*(1, 49) = 12.08, *p* = 0.001, $\eta_{p}^{2}=0.20$. Participants in the WAFA condition also reported believing that the interview had helped them to remember more (*M* = 4.19, *SD* = 0.69) than participants in the Control interview condition (*M* = 3.60, *SD* = 0.71), *F*(1, 49) = 9.11, *p* = 0.004, $\eta_{p}^{2}=0.16$. Participants in the WAFA condition reported feeling more comfortable (*M* = 4.81, *SD* = 0.40) than participants in the Control condition (*M* = 4.44, *SD* = 0.65), *F*(1, 49) = 5.95, *p* = 0.018, $\eta_{p}^{2}=0.11$. There were no significant differences between conditions for mean concentration ratings (*M*_WAFA_ = 4.31, *SD* = 0.68; *M*_Control_ = 4.12, *SD* = 0.60), *F*(1, 49) = 1.09, *p* = 0.302, $\eta_{p}^{2}=0.02$, nor perceived accuracy ratings (*M*_WAFA_ = 3.81, *SD* = 0.66; *M*_Control_ = 3.52, *SD* = 0.77), *F*(1, 49) = 1.98, *p* = 0.167, $\eta_{p}^{2}=0.01$. The final Likert-type question asked participants in the WAFA condition only to rate the utility of the post-it note approach to separating the topics verbalised during the interviews. The mean response was 4.50 (*SD* = 0.76), indicating that participants found them quite useful/very useful.

All participants were asked to rate the pace of the interview (1 = too fast; 2 = too slow; 3 = about right). There was no significant difference between interview conditions, *F*(1, 49) = 0.22, *p* = 0.638, $\eta_{p}^{2}=0.05$, (*M*_WAFA_ = 2.96, *SD* = 0.20; *M*_Control_ = 2.92, *SD* = 0.40), with 96% of participants believing the pace of the interview was about right. All (100%) participants in both conditions reported that the instructions given by the interviewer were clear and that they had understood the instructions given by the interviewer.

## Discussion

Police interviews are formal social interactions where interviewers seek to elicit an accurate and complete retrieval and narration of a past, personally experienced event. Recent evidence has begun to shed light on how autistic individuals’ social-cognitive profile of strengths and weaknesses impacts their ability to give evidence. The CI, which is currently the most prevalent evidence-based technique in the CJS, fails to increase the completeness of autistic witnesses’ accounts and reduces their accuracy, which may be due in part to the lack of explicit parameters concerning their retrieval attempts (e.g. Maras & Bowler, 2010). However, currently there exists no alternative theoretically driven, legally appropriate interview framework to elicit more complete and accurate information about what they have experienced. This is concerning because autistic individuals are disproportionately more likely to be questioned by police than TD individuals (Brown-Lavoie et al., 2014; Chaplin & Mukhopadhyay, 2018; Heeramun et al., 2017; Lindblad & Lainpelto, 2011; Rava et al., 2017; Tint et al., 2017, 2019; Weiss & Fardella, 2018). The aim of this research was to test a novel interview technique that offered autistic individuals support in a way that guided them more concretely through their recall attempts.

Based on the theoretical and empirical literature, and with reference to current best practice guidance for eliciting information from vulnerable witnesses, we developed the WAFA interview technique. The WAFA method enables witnesses to impose an individual parameter-bound structure to their recall by self-segmenting the to-be-remembered event at the outset, before then freely recalling everything they can remember, following which they respond to interviewer prompts within each of these segments. As a first step towards understanding the efficacy of WAFA, we empirically investigated the technique employing a mock witness paradigm in conditions of intentional encoding. We hypothesised that WAFA would improve the quality of the accounts provided by autistic witnesses. Indeed, the completeness of participants’ episodic recall improved significantly, as evidenced by the verbalisation of around 15% more correct information by both autistic and TD witnesses and with a further 6% increase in overall accuracy. Post-interview feedback revealed that participants in the WAFA condition reported the interview had helped them to think harder and remember more, and that they had felt more comfortable. It is promising that both autistic and TD participants performed significantly better in WAFA interviews than control interviews, and that they were more positive about both their performance and the interview process itself.

It is worth noting, however, that while WAFA increased the amount of correct details they reported, autistic witnesses nevertheless recalled fewer correct details overall compared to TD witnesses, even with the WAFA technique. Disentangling whether this reflected poorer memory for the event per se or simply a reduced ability or inclination to report details is beyond the scope of the current study, but this is an important question for future research in order to inform further developments to interview techniques (see Maras et al., 2020, for further discussion on this issue). Future studies should also compare the WAFA technique directly with the CI to establish whether it might generally be superior for both autistic and TD witnesses, or whether it primarily supports autistic witnesses relatively more effectively when compared to the alternative CI technique.

There are several possible explanations as to why WAFA interviews were effective in improving the completeness and accuracy of autistic as well as TD witnesses’ testimony. First, the initial instruction to retrieve individual topics or sub-events within the videos may reduce demands on relational retrieval processes, which would typically aid the reconstruction of the global narrative of the event in terms of the relations between individual event details (who did what to whom, where, when and how) and which are a source of difficulty for autistic individuals (Bowler et al., 2009; Gaigg & Bowler, 2018; Gaigg et al., 2008). The WAFA technique reduces demands on relational processing by assisting participants in generating the overarching event segments from which to recall details. In contrast, the mental context reinstatement procedure of the CI assumes that environmental cues can facilitate participants’ retrieval of both details and broader event segments via a bottom-up associative network, for example, relating to perceptions, emotions, persons, places and actions, which are then used to reconstruct an entire memory (see Dando, 2013; Geiselman & Fisher, 2014).

Second, asking witnesses to recall details within each of the ‘topic boxes’ that are generated in WAFA should reduce demands on executive processes, since participants have available their self-generated event structure that they would otherwise need to hold in mind during recall. Only event details within a single sub-event or topic box are recalled at any one time, and the post-it notes serve as a visual reminder of the rest of the event structure (to be recalled in separate efforts), thus freeing up executive resources to engage in a detailed retrieval process (see Maister et al., 2013). Future research that examines whether executive functioning predicts performance under the different interview techniques would be helpful to illuminate this issue further, and aid in the further development and refinement of techniques.

Third, witness-generated segmentation of an event into its component parts is consistent with witness-compatible questioning (e.g. MacDonald et al., 2017) in that it provides scaffolding for the individual processing styles of autistic (and indeed TD) witnesses (e.g. Pellicano & Burr, 2012) and allows the interviewee to revisit topics in the order that they have first recalled them. Paulo et al. (2016, 2017) recently developed an additional component of the CI whereby witnesses are explicitly instructed to organise their episodic recall semantically rather than temporally, on the basis that recalling a crime event in category clusters may be more compatible with an individual witness’ mental organisation of the event. Following free recall, witnesses are then instructed to recall everything they can remember, focusing on just one category of information at a time (e.g. objects, locations, people, etc.). While category clustering recall has been shown to elicit more correct details from TD witnesses (e.g. Paulo et al., 2016, 2017; Thorley, 2018), the interviewer directs the nature and order of categories to be recalled and it is preceded by unbound free recall, which is problematic for autistic witnesses. The WAFA interview, in contrast, utilises a similar principle of category clustering but these categories are events rather than types of details and are determined by the witness rather than the interviewer at the outset.

Finally, by explicitly segmenting the event and revisiting each of the self-directed topics in turn (with a visual schedule in the form of the post-it notes), WAFA may reduce implicit task demands, alleviating the need to infer what and how much to recall, as is often the case during an unbound, unstructured free recall attempt of an entire event (see Kenworthy et al., 2008; Müller et al., 2008; see also White et al., 2009).

The present findings also revealed that removing the narrative structure of an event had a profound effect on recall – diminishing both accuracy and completeness across all detail types. In contrast to our initial predictions, both the autistic and TD groups’ overall recall was similarly negatively impacted when the event’s narrative structure was lost, indicating a lack of group differences at the encoding stage. While there is robust evidence that the comprehension and production of narratives can be difficult for autistic individuals (e.g. Boucher, 1981; Diehl et al., 2006; Hilvert et al., 2016; Kuijper et al., 2017; Lee et al., 2018; Losh & Capps, 2003; Loveland et al., 1990; McCabe et al., 2013), there is also some limited evidence suggesting that autistic individuals can sometimes utilise narratives to enhance their encoding and subsequent retrieval. For example, in contrast to previous findings of no enhancement of emotionally arousing content on recall of static stimuli such as words (Gaigg & Bowler, 2008), sentences (Beversdorf et al., 1998) and images in ASD (Deruelle et al., 2008), over two experiments by Maras et al. (2012) found that emotionally arousing stories were remembered better (and forgotten less) than neutral events by both autistic and TD participants. Maras et al. concluded that autistic participants may have utilised the clear narratives of the arousing event stimuli used in the study to strengthen their retrieval (see also Miller et al., 2014). The present findings of more complete and accurate recall of unscrambled videos provide more marked evidence that both TD and autistic individuals do spontaneously use an event’s narrative and natural segmentation in actions, locations and semantic changes to bolster their memory (but see Zalla et al., 2013).

Limitations of the present study are acknowledged. While recall was coded for completeness and accuracy, narrative coherence was not assessed. Thus, it is unclear whether WAFA interviews improved the ability of autistic witnesses to provide more coherent and relevant narratives – which is an important avenue for future research given the substantial evidence of differences in narrative ability in ASD, which in turn may impact perceptions of credibility (e.g. Crane et al., 2018). It is also important to note that the present study utilised a relatively short delay of around 30 min; in real life, it is unlikely that a witness would receive a formal investigative interview this soon after witnessing a crime. Nonetheless, the current findings demonstrate that autistic witnesses can provide testimony that is accurate as TD witnesses when interviewed shortly after the event, highlighting the importance of conducting witness interviews as soon as possible (see also Almeida et al., 2019a, 2019b). If anything, the benefit of WAFA may be even greater with longer delays, but future research should examine this, alongside its impact on account coherence. Furthermore, there were no IQ or working memory score differences between the autistic and TD groups – which was important to ensure that any observed group differences in performance were attributable to diagnostic status (Burack et al., 2004) – however, this does limit the generalisability of the findings to autistic individuals with accompanying intellectual disability.

This research is timely because there is an urgent need for evidence-based guidance for CJS professionals on how to interview autistic witnesses. The present findings indicate that gathering a WAFA whereby the witness self-segments events first, before re-visiting each of the topics in detail in the order they were recalled, is a promising technique to elicit a more detailed and accurate account of witnessed events – for both autistic and TD witnesses. This technique may also be useful outside of the CJS, from clinical practice to employment interviews. Future work should explore this in more depth, with different types of episodic and autobiographical events.

## Appendix 1

### Post-interview feedback questions

**1. Please explain what you liked about the interview**
**2. Please explain what you did not like about the interview**
**3. Did the interview instructions help you to concentrate?** (please circle one)

**Table:**

Not at all	Not much	Neutral	Quite well	Very well
1	2	3	4	5

Please explain your answer

**4. How well do you feel the interview helped you to think hard about the video?** (please circle one)

**Table:**

Not at all	Not much	Neutral	Quite well	Very well
1	2	3	4	5

Please explain your answer

**5. How well do you feel the interview helped you to remember much of what you saw in the video clips?**

**Table:**

Not at all	Not much	Neutral	Quite well	Very well
1	2	3	4	5

Please explain your answer

**6. How well do you feel the interview helped you to recall an accurate account of what you saw in the video clips, with few mistakes?** (please circle one)

**Table:**

Not at all	Not much	Neutral	Quite well	Very well
1	2	3	4	5

Please explain your answer

**7. Were the instructions given to you by the interviewer clear?** (please circle one)

Yes   No

**8. Did you understand the instructions given to you by the interviewer?** (please circle one)

Yes   No

**9. If you did find any of the instructions confusing or difficult please explain which instructions you found difficult**
**10. What could the interviewer could have done to help you remember more?**
**11. Did you feel the pace of the interview was?** (please circle one)

*too fast  too slow  about right*

Please explain your answer

**12. How comfortable did you feel with the interviewer’s manner or questioning style?** (please circle one)

**Table:**

Very uncomfortable	Quite uncomfortable	Neutral	Quite comfortable	Very comfortable
1	2	3	4	5

Please explain your answer

**13. (WAFA only) How useful did you find the post-it notes that separated out the different things you remembered from the video useful?** (please circle one)

**Table:**

Not at all	Not much	Neutral	Quite useful	Very useful
1	2	3	4	5

Please explain your answer

## Notes

1. From here on, the term ‘witness’ is used throughout to encompass both third party witnesses and victims who, through providing evidence in the Criminal Justice System, become a witness.

2. The stimulus videos are available online at: https://www.youtube.com/playlist?list=PLV9WU7h_aldknW3zmWzrgMO7rEBtNrfVD
